# ZooKeys 150: Three and a half years of innovative publishing and growth

**DOI:** 10.3897/zookeys.150.2431

**Published:** 2011-11-28

**Authors:** Terry Erwin, Pavel Stoev, Teodor Georgiev, Lyubomir Penev

**Affiliations:** 1 Smithsonian Institution, Washington, DC, USA; 2 Bulgarian Academy of Sciences & Pensoft Publishers, Sofia, Bulgaria; 3 Pensoft Publishers, Sofia, Bulgaria

‘ZooKeys publishes articles of the future’

Roderic Page, title of a blog post in iPhylo

On the 28^th^ of November 2011, the open access journal ZooKeys published its 150th issue – an excellent occasion for the Editorial team to evaluate the journal’s development and its position among systematic biology journals worldwide.

From the very beginning, ZooKeys was designed as an innovative journal aiming at developing new methods of publication and dissemination of taxonomy information, including publishing of atomized, semantically enhanced automated exports to global data aggregators, such as Encyclopedia of Life (EOL), the Global Biodiversity Information Facility (GBIF), Plazi, Species-ID and others. Since its launch on the 4^th^ of July 2008, the journal provided registration of all new taxa and authors in ZooBank on a mandatory basis and continues to include their Life Science Identifiers (LSID) in the published articles (Penev et al. 2008). Also since its first issue, ZooKeys made it a routine practice of supplying all new taxa to the Encyclopedia of Life through XML mark up. In the subsequent years, the journal joined GBIF and the Taxonomic Databases Working Group (TDWG) in the development of common data publishing standards and workflows.

In 2009, ZooKeys initiated several pilot projects thereby setting foundations of semantic tagging of, and enhancements to, biodiversity articles using the TaxPub XML schema, an extension of the DTD (Document Type Definitions) of the National Library of Medicine (USA) (Penev et al. 2009a; Catapano 2010). The first one was the milestone article ‘The symphytognathoid spiders of the Gaoligongshan, Yunnan, China’ (Miller et al. 2009) where, for the first time in systematic zoology, a unique combination of data publication and semantic enhancements was applied within the mainstream process of journal publishing. The article demonstrated how all primary biodiversity data underlying a taxonomic monograph could be published as a dataset under a separate DOI within the paper and the occurrence dataset could be integrated and accessed through GBIF data portal simultaneously with the publication. In the same year, data publication practices of online identification keys (Penev et al. 2009b) were exemplified by the pioneering articles of Sharkey et al. (2009) and that was shortly followed by others (van Noort and Johnson 2009; Stoev et al. 2010).

On the 30^th^ of June 2010, ZooKeys published a special issue ‘Taxonomy shifts up a gear: New publishing tools to accelerate biodiversity research’ which marked the journal’s brand new innovative publishing model, based on XML editorial workflow and on the TaxPub XML schema. From that time on, ZooKeys has been published in four formats – full-colour print version, PDF, HTML, and XML (Penev et al. 2010a). This happened simultaneously with the implementation in the editorial process of the Pensoft Mark Up Tool (PMT), a program specially designed for XML tagging and semantic enhancements (Penev et al. 2010b). Four papers using three different types of manuscript submission (Stoev et al. 2010; Blagoderov et al. 2010; Brake and Tschirnhaus 2010; Taekul et al. 2010) were used to exemplify the process.

Realizing the importance of Wiki environment for popularization and dissemination of the biodiversity data, in April 2011 ZooKeys undertook another major step towards its modernization. Three sample papers (Hendriks and Balke 2011; Stoev and Enghoff 2011; Bantaowong et al. 2011) demonstrated the automated integration of species descriptions at the day of publication to Species-ID – an open access Wiki-based resource for biodiversity information. This was achieved by programming a special tool, named Pensoft Wiki Convertor (PWC), which transforms the XML versions of the papers into MediaWiki-based pages (Penev et al. 2011a).

In October 2011, ZooKeys launched its *multiple-choice model* for publishing biodiversity data that provides a non-exclusive choice of mechanisms for the publication of data of different kinds and complexity, in cooperation with specialized data repositories and data aggregators, based on the previously published Pensoft Data Publishing Policies and Guidelines for Biodiversity Data (Penev et al. 2011b) One of the most important steps in this direction was the launch of an innovative route for publishing occurrence data and taxon checklists using an approved TDWG standard (Darwin Core), enriched metadata descriptions for the published datasets, and the possibility of downloading both data and metadata in a machine-readable form, the so-called Darwin Core Archive. This is supported by a specialized tool of GBIF, the Integrated Publishing Toolkit (IPT). Use of this tool allows the production of so-called “Data Paper” manuscripts that formally describe a dataset’s metadata as a peer-reviewed and citable scholarly publication (Chavan and Penev in press).

A second important element of the *multiple-choice* data publishing model of ZooKeys was the integration of its data publishing workflow with the Dryad Digital Repository, thus providing an option to its authors to archive data files of different kinds and complexity (e.g., phylogenetic, morphometric, ecological, environmental, etc.).

The latest innovation of ZooKeys was announced just a few days before publication of this editorial. On the 22nd of November 2011, ZooKeys launched an automated export and indexing of identification keys metadata published in the journal in KeyCentral – a global database of keys and other identification resources for living organisms.

**Figure 1.**
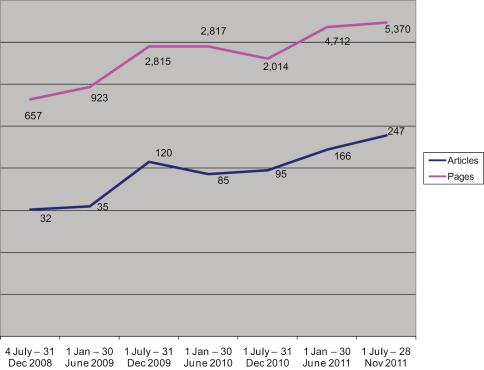
Total number of published articles and pages on six-month intervals.

ZooKeys has shown a significant publication growth for the 41 months of its existence (Fig. 1). Starting with a mere 32 articles in 2008, the journal has rapidly increased its production to 180 in 2010 and 413 in 2011 (through the 28^th^ of November). Likewise the number of published pages has grown from 657 in 2008, 3,738 in 2009, 4,831 in 2010 to 10,082 in 2011. The growth rate for 2011 in comparison to 2010 in the number of published pages is more than 100% and will most probably exceed 120% by the end of the year. For three and a half years, ZooKeys has published overall 19,308 pages (780 articles), a figure that is comparable to the number of pages published by Zootaxa during its first 41 months of activity (16,738 pages – see Zhang 2011 and http://www.mapress.com/zootaxa).

Altogether, 1,558 new species-group, 192 new genus-group and 16 new family-group taxa have been published in the journal since its launch (Table 1). This makes overall 1,766 new taxa in total, or 43 new taxa per month on average. Comparing these figures with the Index of Organism Names of Zoological Record (accessed 18 November 2011) ZooKeys has published approximately 2.5% of all the 69,224 new animal taxa described from 2008 to 2011, and ranks second (immediately after Zootaxa) in the top 10 journals publishing new taxa. The data retrieved from ZooBank show that one third of all new names registered in ZooBank since June 2008 have been published in ZooKeys. The total number of ZooKeys authors registered in ZooBank up to issue 148 reached 754 (Richard Pyle, in litt.).

**Table 1.** New taxa published in ZooKeys that have been registered and assigned LSIDs in ZooBank (data for issues 1-148 provided by Richard Pyle, in litt.).

**Categories**	**Number**
Species-group names	1,558
Genus-group names	192
Family-group names	16
**Total**	**1,766**

**Figure 2.**
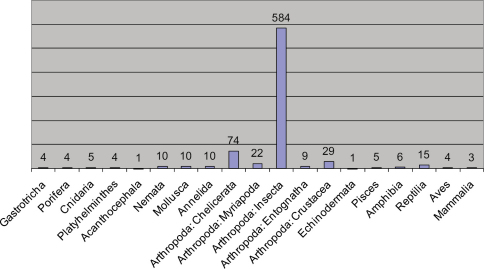
Distribution of the published articles per taxon.

Figure 2 summarizes the distribution of articles per large taxon. Unsurprisingly, the highest number of articles published in ZooKeys dealt with insects (584). The articles on Coleoptera (249) dominate and together with those dealing with Hymenoptera (122) make up approximately 48% of all ZooKeys articles. Those on Lepidoptera (77), Hemiptera (42) and Diptera (39) also form a significant share of the published volumes. Among the non-insect invertebrates the highest number of articles were published on Chelicerata (74), followed by Crustacea (29) and Myriapoda (22). The total number of articles dealing with vertebrates is comparatively low (33), nearly half of them refer to reptiles (15).

The top 10 most accessed ZooKeys papers through the 20^th^ of November 2011 are listed in Table 2. The 972 page monograph of Bouchard et al. (2011) ‘Family-Group names in Coleoptera (Insecta)’ is taking the first place reaching 8,623 page views on the 20^th^ of November. In the top 3 most viewed articles are also the ‘Data publication and dissemination of interactive keys’ (Penev et al. 2009) and ‘Cretaceous Crocodyliforms from the Sahara’ (Sereno and Larsson 2009), with 7,716 and 6,275 page views, respectively.

**Table 2.** Top ten most viewed articles of ZooKeys (according to the ZooKeys website counter accessed on the 20^th^ of November 2011).

**Article**	**Page views**
Bouchard et al. 2011 – Family-Group names in Coleoptera (Insecta)	8,623
Penev et al. 2009 – Data publication and dissemination of interactive keys under the open access model	7,716
Sereno and Larsson 2009 – Cretaceous Crocodyliforms from the Sahara	6,275
Baldwin et al. 2011 – Seven new species within western Atlantic *Starksia atlantica*, *Starksia lepicoelia*, and *Starksia sluiteri* (Teleostei, Labrisomidae), with comments on congruence of DNA barcodes and species	5,283
Achterberg and Long 2010 – Revision of the Agathidinae (Hymenoptera, Braconidae) of Vietnam, with the description of forty-two new species and three new genera	5,107
Hendrich and Balke 2011 – A simultaneous journal / wiki publication and dissemination of a new species description: *Neobidessodes darwiniensis* sp. n. from northern Australia (Coleoptera, Dytiscidae, Bidessini)	3,986
Hong et al. 2011 – A revision of the Chinese Stephanidae (Hymenoptera, Stephanoidea)	3,888
Wizen and Gasith 2011 – Predation of amphibians by carabid beetles of the genus *Epomis* found in the central coastal plain of Israel	3,818
Heads and Leuzinger 2011 – On the placement of the Cretaceous orthopteran *Brauckmannia groeningae* from Brazil, with notes on the relationships of Schizodactylidae (Orthoptera, Ensifera)	3,655
Murphy et al. 2011 – The dazed and confused identity of Agassiz’s land tortoise, *Gopherus agassizii* (Testudines: Testudinidae) with the description of a new species and its consequences for conservation	3,653

In order to increase public awareness to the importance of taxonomy and biodiversity studies in general, in May 2011 Pensoft opened a press office and started active public relations (PR) activities. Authors are invited to draft press releases on their findings at the moment of acceptance of their publications. The Pensoft PR team offers support to the authors in “translating” the technical texts into a language that would be of interest for the public. Press releases are posted to a number of sites; the first place, EurekAlert!, is the world largest online distributor of science news supplying information to more than 7,500 mass media and independent science journalists. A list of the top 10 most accessed press releases of ZooKeys articles is given in Table 3. The press release on the new Late Cretaceous family of wasps, Plumalexiidae, described in a Festschrift honouring the Russian paleontologist Alexandr Rasnitsyn has hitherto attracted the highest attention in the world media. Of similar high popularity in the world news outlets was the unique observation of oviposition behaviour of four ant parasitoids that was filmed for the first time and movies uploaded in YouTube (Durán and Achterberg 2011). Another ZooKeys article showing *Epomis* beetles preying on amphibians (Wizen and Gasith 2011) whhose associated movies were posted on YouTube have been watched 344,325 times in 6 months! This is further evidence that taxonomic discoveries enjoy a lot of interest from the public, if they are properly and attractively distributed.

**Table 3.** Top 10 most accessed press releases of ZooKeys articles posted through EurekAlert! (from the EurekAlert! counter). The counter registers only the downloads from EurekAlert! mostly by science media and journalists. The actual number of readers may actually be much higher than this number.

**Title**	**Author/s and year of publication of the original article**	**Date posted**	**Page views since posted**
New family of wasps found in North American amber, closest relatives in southern hemisphere	Brothers 2011	26-Sep-2011	3,412
Death from above: Parasite wasps attacking ants from the air filmed for the first time	Durán and Achterberg 2011	29-Aug-2011	2,749
A living species of aquatic beetle found in 20-million-year-old sediments	Fikáček et al. 2011	6-Oct-2011	2,676
Chinese researchers identify insect host species of a famous Tibetan medicinal fungus	Wang and Yao 2011	8-Sep-2011	2,340
Small insects attacks and kill amphibians much bigger than themselves	Wizen and Gasith 2011	20-May-2011	2,309
A new species of fossil silky lacewing insects that lived more than 120 million years ago	Peng et al. 2011	5-Oct-2011	2,203
Jewel beetles, obtained from local people, turn out to be 4 species unknown to science	Bílý and Nakládal 2011	7-Jul-2011	1,921
A new species of a tiny freshwater snail collected from a mountainous spring in Greece	Radea 2011	1-Nov-2011	1,885
Unknown species and larval stages of extremely long-legged beetles discovered by DNA test	Freitag and Balke 2011	18-Oct-2011	1,437
Earliest psychomyiid caddisfly fossils, from 100-million-year-old Burmese amber	Wichard et al. 2011	5-Oct-2011	1,350

ZooKeys represents a new type of a journal whose mission is to create new horizons for taxonomists through modern technology and widespread promulgation of biodiversity data. Thanks to its continuously applied innovations, and especially owing to the commitment of its professional editorial team, the journal will continue to facilitate and accelerate biodiversity research at the same pace, along with its sister journals PhytoKeys and MycoKeys. We sincerely thank all editors and reviewers for their selfless support and professional editorial work, as well as our hundreds of friends and colleagues that have been actively discussing with us and sharing their opinions on the ‘ZooKeys’ project throughout the years. Without your kind assistance the journal would never have become as popular as it is now and would never merit its consideration as one of the most technologically advanced journals in biological science.
